# Mechanisms and prospects of circular RNAs and their interacting signaling pathways in colorectal cancer

**DOI:** 10.3389/fonc.2022.949656

**Published:** 2022-08-03

**Authors:** Shuwei Wang, Liang Cheng, Haotian Wu, Gan Li

**Affiliations:** Department of General Surgery, Wuxi Affiliated Hospital of Nanjing University of Chinese Medicine, Wuxi, China

**Keywords:** colorectal cancer, circular RNA, signaling pathway, biological function, clinical value

## Abstract

Colorectal cancer (CRC) is the leading malignant tumor in terms of morbidity and mortality worldwide, and its pathogenesis involves multiple factors, including environment, lifestyle, and genetics. Continuing evidence suggests that circular RNAs (circRNAs), as a novel non-coding RNA, constitute an important genetic variable in the pathogenesis of CRC. These circRNAs with covalently closed-loop structures exist objectively in organisms. They not only have the biological functions of regulating the expression of target genes, changing the activity of proteins, and translating proteins, but also play a key role in the proliferation, invasion, migration, and apoptosis of tumor cells. CRC is one of the most common cancers in which circRNAs are involved in tumorigenesis, metastasis, and drug resistance, and circRNAs have been demonstrated to function through crosstalk with multiple signaling pathways. Therefore, this review summarizes the biological and carcinogenic functions of circRNAs and their related PI3K/AKT, MAPK, Notch, JAK/STAT, Hippo/YAP, WNT/β-catenin, and VEGF signaling pathways in CRC. We further explore the clinical value of circRNAs and important signaling proteins in the diagnosis, prognosis, and treatment of CRC.

## Introduction

Colorectal cancer (CRC) is the third most commonly diagnosed cancer and the second leading cause of cancer deaths worldwide, with an estimated 1.9 million new cases and 935,000 deaths in 2020 (1). The incidence of CRC has stabilized and declined in highly developed countries as a result of national screening programs and colonoscopy (2). But with economic progress in developing countries, the number of new CRC cases worldwide is expected to reach 2.5 million by 2035 (3). At present, the 5-year survival rate of patients with early CRC is close to 90% (4). However, among newly diagnosed patients with CRC, 20% have already had metastasis, and another 25% will develop metastasis due to locally advanced tumors (5). Moreover, metastatic CRC has a poor prognosis, with a 5-year survival rate of less than 20% (6). Therefore, to seek effective biomarkers for early diagnosis of CRC and new therapeutic targets for advanced and recurrent CRC, it is necessary to further explore and clarify the molecular mechanisms underlying the development and metastasis of CRC.

Circular RNAs (circRNAs) are newly discovered non-coding RNAs (ncRNAs) that exist objectively in living organisms (7). These circRNAs have a covalently closed-loop structure, missing the 5′-3′ terminals and polyadenylate tails (8). The development of high-throughput RNA sequencing and bioinformatic tools has successfully detected thousands of circRNAs distributed in a variety of tissues, cell types, and biological fluids (9, 10). Moreover, researchers have revealed that these RNAs have cell-specific, tissue-specific, and time-specific expression patterns and are conserved across species (11, 12). Recent evidence indicates that circRNAs are not only significantly associated with neurological disorders, cardiovascular diseases, and autoimmune diseases (13–15), but also play a regulatory role in cancer-related processes such as tumorigenesis, progression, and cell apoptosis (16–18). CRC is one of the most commonly reported cancers in which circRNAs are involved in tumorigenesis and metastasis (18–20).

Signal transduction is a common way to regulate basic cellular processes in humans, and abnormal regulation of signal transduction can lead to the occurrence of pathological states such as cancer and autoimmunity (21, 22). It is widely believed that circRNA promotes cancer cell proliferation and metastasis by interacting with key components of major signaling pathways (23, 24). In CRC, the reported crosstalk signaling pathways with abnormally expressed circRNA include phosphatidylinositol 3-kinase (PI3K)/AKT (25), mitogen-activated protein kinases (MAPK) (26), Notch (27), Janus kinase/signal transducers and activators of transcription (JAK/STAT) (28), Hippo/YAP (29), WNT/β-catenin (30), and vascular endothelial growth factor (VEGF) (31). In this review, we summarized the molecular mechanism and role of circRNAs and related signaling pathways in the occurrence and progression of CRC by searching for keywords in Pubmed, Web of Science, ScienceDirect, and Springer SLCC databases. The clinical application value of circRNAs in the diagnosis, prognosis, and treatment of CRC was further discussed.

## Genetic and transcriptional characterization of CRC

Recent research suggests that most CRC cells originate from stem cells or stem cell-like cells (32). The accumulation of multiple genetic and epigenetic changes produces these cancer stem cells (CSCs), which ultimately activate oncogenes and inactivate tumor-suppressor genes (33, 34). Two major precursor pathways represent multiple genetic and epigenetic events in a fairly continuous sequence. Most CRCs are chromosomal instability sequences (70-90%), also known as the traditional adenoma-carcinoma pathway (35). In this pathway, tumor development is caused by the sequential accumulation of mutations in the WNT, epidermal growth factor receptor (EGFR), P53, and transforming growth factor-beta (TGF-β) signaling pathways (36). Another is the serrated tumor pathway, which involves activating BRAF mutations or DNA mismatches to repair gene inactivation and accounts for 10-20% of CRCs (37, 38).

In 2015, the International CRC Subtype Consortium proposed a more comprehensive transcriptome classification based on gene expression profiles (39). CRC is divided into four consensus molecular subtypes (CMS): CMS1 (MSI immunity, 14%), CMS2 (canonical, 37%), CMS3 (metabolic, 13%), and the subtype with the worst prognosis, CMS4 (mesenchymal, 23%) (40). These CMS group classifications embody markedly different molecular characteristics associated with biological and clinical stratification and are the basis for targeted interventions (41). There are differences not only in embryology, anatomy, and biology but also in molecular characteristics between right colon cancer (hepatic curvature of transverse colon, ascending colon, and cecum) and left colon cancer (splenic curvature of transverse colon, descending colon, and sigmoid colon) and the rectum (42). Right colon cancer is more common in CMS1 and CMS3 subtypes, while left colon cancer is predominantly in CMS2 subtypes (43).

## The biological and oncogenic functions of circRNAs in CRC

**Figure 1:**
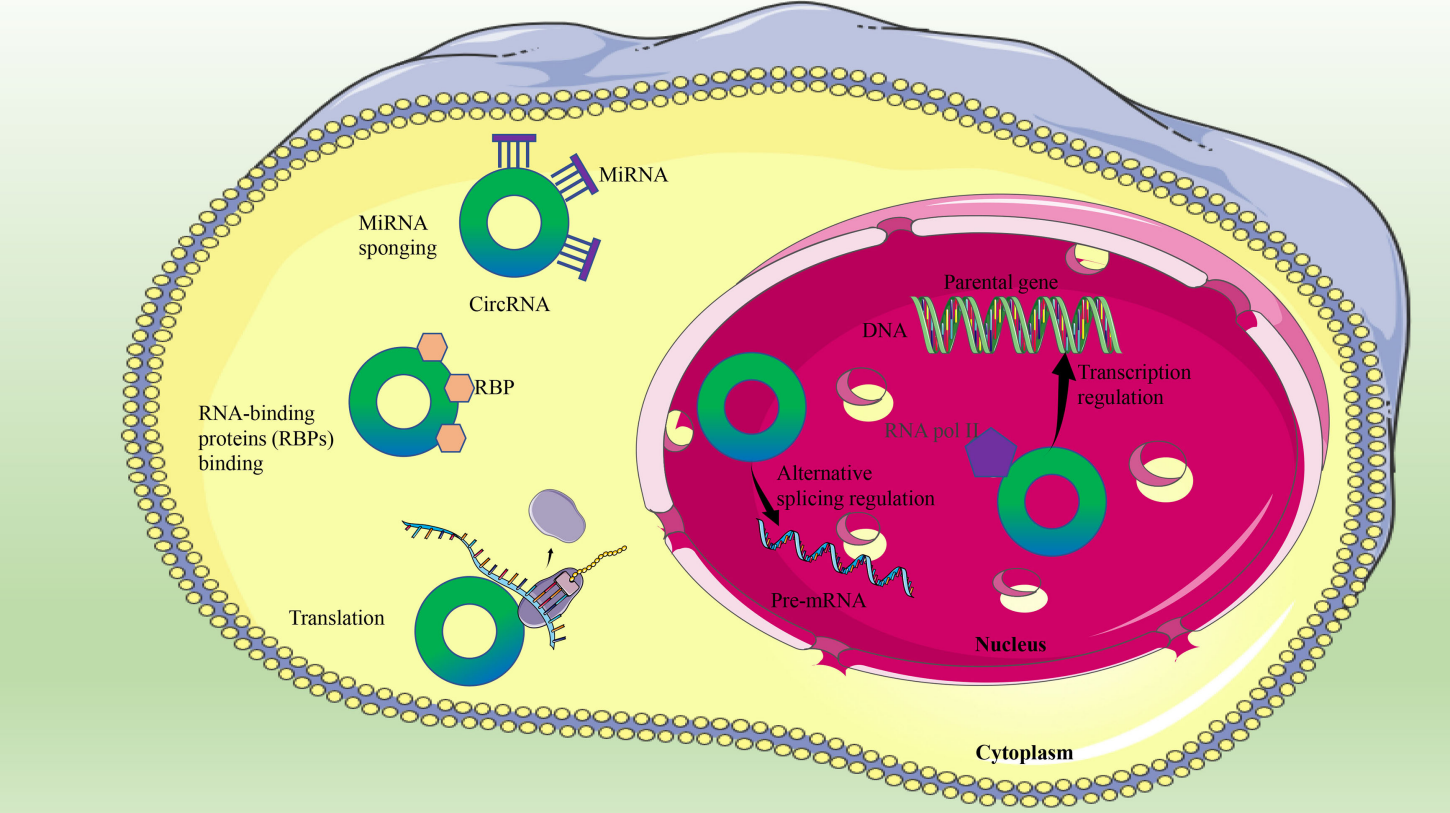
The biological functions of circRNAs in CRC.

CircRNAs play different biological functions according to their localization in the cytosol or nucleus (44). First of all, circRNAs can regulate gene transcription and alternative splicing, and nuclear circRNAs can also induce parental gene expression (45). Second, some circRNAs, such as circ_0128846 (29) and circ_0106714 (46), influence the expression of target genes by competitively binding microRNA (miRNA) or acting as miRNA sponges, which is the most widely studied mechanism of circRNA in the progression of CRC (47). In addition, another biological function of circRNAs is that their interactions with RNA-binding proteins cause changes in protein activity (48). Recently, two novel circRNAs (circ-BCL2L12-1 and circ-BCL2L12-2) with different protein binding sites have been identified in CRC. Moreover, circ-BCL2L12-1 overexpression was related to shorter OS, while circ-BCL2L12-2 expression was negatively related to TNM staging in CRC (49). Finally, circRNA has the biological function of translating proteins (50), and circ-PPP1R12A is reported to have translation capabilities in CRC (51) (**Figure 1**).

A large number of studies have shown that circRNA can play an important role in the tumor progression of CRC by regulating tumorigenesis transcription factors or oncogene expression (52). In the study of Li’s team, 448 circRNAs with abnormal expression in CRC were detected by high throughput RNA sequencing. Further *in vitro* experiments showed that the down-regulation of circDDX17 could enhance the proliferation, invasion, and migration of CRC cells (53). *In vivo* experiments on nude mice have also confirmed that some circRNAs, such as circ-ERBIN, can promote tumor growth and metastasis of CRC (54). Advanced metastatic CRC is the leading cause of cancer-related death. Epithelial-mesenchymal transition (EMT) is a cellular reprogramming process in which epithelial cells acquire a mesenchymal phenotype, which promotes the development of migrating and invading cells (55). Through GEO data set analysis, circ_101951 was found to be a novel circRNA overexpressed in CRC tissues. In-depth studies on its biological function and mechanism showed that circ_101951 could facilitate the migration and invasion of CRC cells by regulating EMT (56). CSCs are subsets of small cells in tumors that drive tumor progression and metastasis (57). Continuing studies have shown that CSCs in CRC are inherently resistant to treatment and are closely related to cancer regeneration and recurrence after conventional treatment (58). Rengganaten et al. revealed circ_0066631 and circ_0082096 as two abnormally expressed circRNAs in CSC-rich CRC globular cells that play an important role in regulating the stemness properties of CSCs (59).

## The role of major signal pathways interacting with circRNAs in CRC

### CircRNA/MAPK signaling axis in CRC

**Figure 2:**
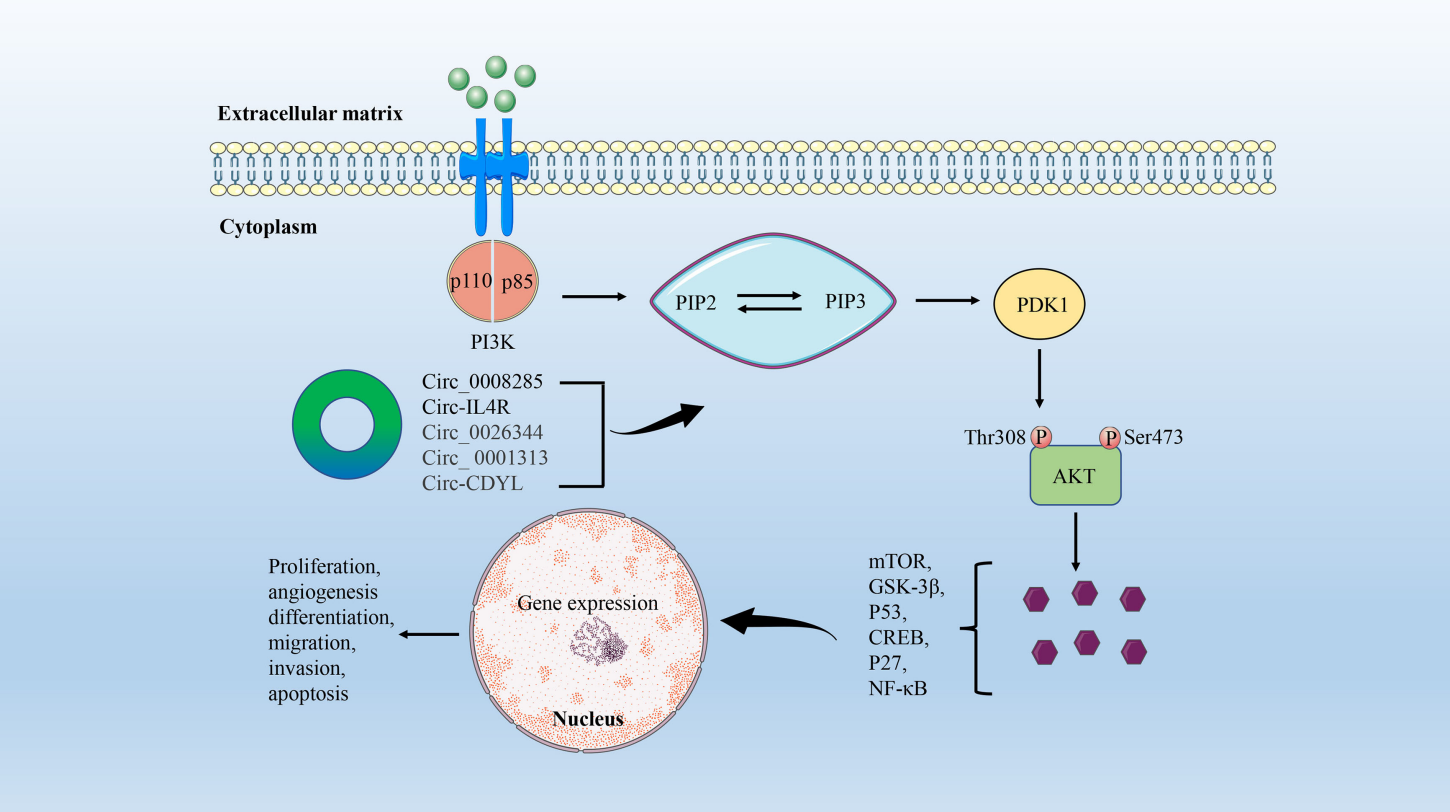
The circRNA/MAPK signaling axis in CRC. This signaling pathway is initiated by the binding of activated growth factors, such as epidermal growth factor (EGF), to tyrosine kinase receptors on the cell surface. This causes the downstream RAS to increase the GTP binding state. RAS-GTP dimers recruit RAF or RAF/MEK dimers to the plasma membrane and promote the activation of RAF and the formation of MEK homologous dimers. This is followed by activation and phosphorylation of downstream ERKs. Finally, ERKs induce phosphorylation of transcription factors and other substrates to participate in cell proliferation, differentiation, migration, and apoptosis in the nucleus.

Compared with other intracellular signaling pathways, the MAPK pathway is more important in cell proliferation, differentiation, migration, apoptosis, and angiogenesis. The most critical signal cascade reaction in all MAPK signal transduction pathways is RAS/RAF/MEK/ERK (60). The pathway is initiated by activation of the RAF kinase family (ARAF, BRAF, and CRAF [RAF1]) by members of the RAS family. Activated RAF protein phosphorylates MEK1/2 and then activates and phosphorylates ERK. Finally, ERKs induce phosphorylation of a variety of substrates, such as transcription factors, which are involved in regulating a variety of cellular functions (61) (**Figure 2**). Abnormal activation of the MAPK signaling pathway in CRC has been reported to occur through activation mutations of RAS and BRAF (62), which are associated with treatment resistance in patients with metastatic CRC (mCRC) (63). Approximately 90% of BRAF mutations are in the V600E series, and although BRAF^V600E^ mutations are rare in CRC (about 10%), their role is important (64). BRAF^V600E^ series mutations in CRC are more common in older women (over 70 years old), right colon tumors, poorly differentiated tumors, and mucous subtypes, and also have a higher frequency of peritoneal metastasis (65). This also predicted poor clinical prognosis (median OS of 11 months) and poor standard treatment response in CRC with BRAF^V600E^ series mutations (66). RAS family is one of the most frequently mutated families in CRC (67). About 40% of mCRCs carry KRAS mutations, mainly in exons 2 (codon 12, 13), 3 (codon 59-61) and 4 (codon 117, 146). Mutations at different points cause different clinical, pathological, and molecular features. Although mutations in NRAS account for only 4% of mCRC, they have similar clinical and pathological features to KRAS mutations. While HRAS mutations are 1%, very few studies have (65, 68).

In CRC, circRNA regulation of the MAPK signaling pathway is a topic widely discussed and worthy of study, although there are few relevant research results at present. It is generally believed that the phosphorylation of MAPK14, the core molecule of the MAPK pathway, by upstream signal kinase kinase 3/6 (MKK3/6) promotes nuclear translocation and promotes the progression of CRC (69). Based on the above results, Wang et al. further verified that circ_0131663 (circ-MAPK14) can reduce the nuclear translocation of MAPK14 by competitive binding with upstream MKK6 through a peptide encoding 175 amino acids, and ultimately inhibit the progression and metastasis of CRC (70). In addition, the functions and mechanisms of other novel circRNAs in CRC have also been preliminarily explored. Circ-ITGA7 was significantly underexpressed in CRC tissues and cell lines, and it was found by functional experiments that the expression of circ-ITGA7 prevented the growth and metastasis of CRC cells *in vitro* and *in vivo*. Further mechanistic studies have shown that circ-ITGA7 inhibits the growth and metastasis of CRC tumors by inhibiting RAS/RAF/MEK/ERK signaling pathways and promoting ITGA7 transcription (71). Another novel circRNA, CIRS-7, acts as a competitive endogenous RNA (ceRNA) of miR-7 to regulate EGFR/RAF1/MAPK signal transduction and plays an important role in CRC progression (72).

### CircRNA/PI3K/AKT signaling axis in CRC

**Figure 3:**
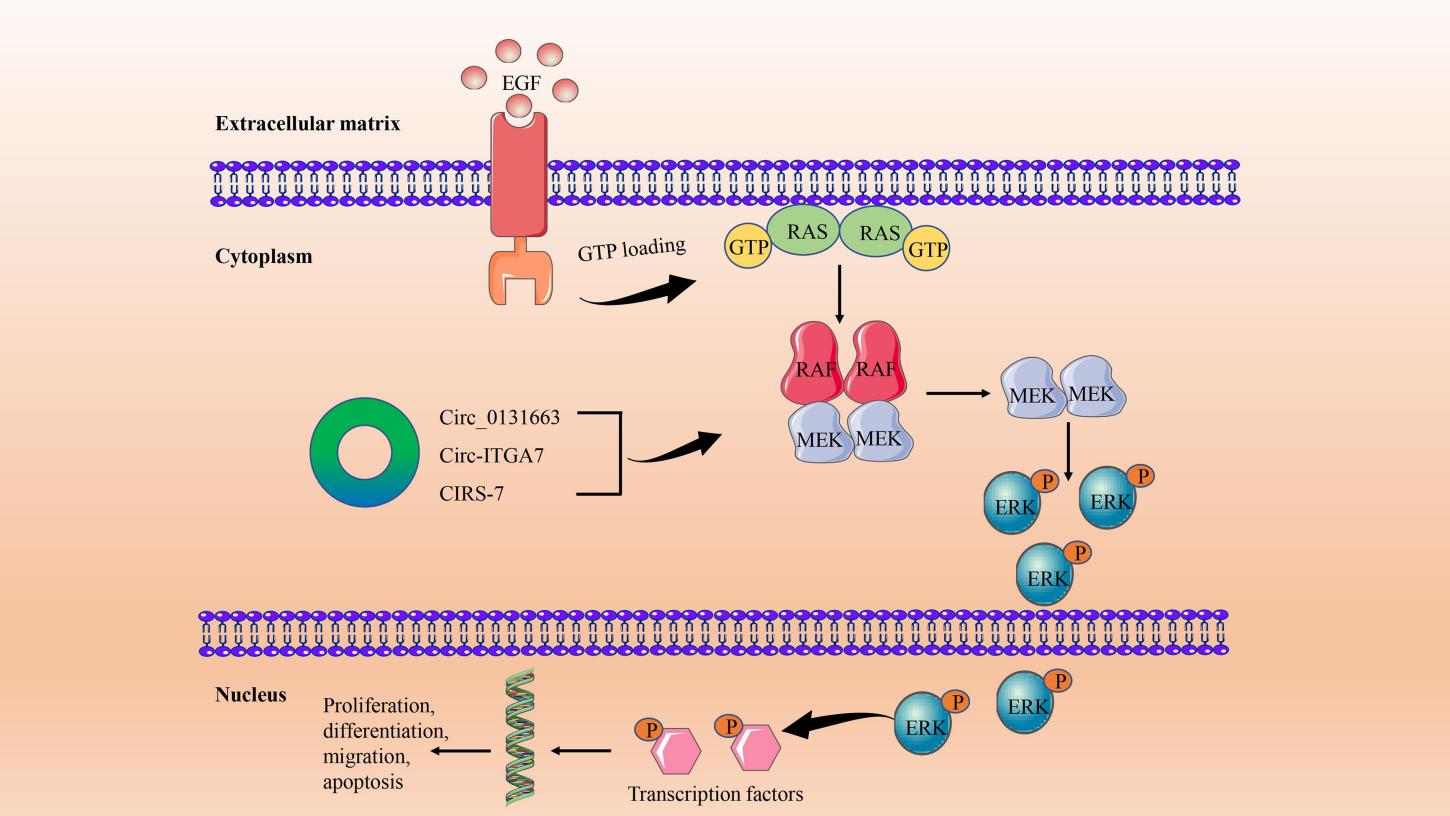
The circRNA/PI3K/AKT signaling axis in CRC. PI3K consists of a catalytic (P110) domain and a regulatory (P85) domain. PI3K is activated by a variety of growth factors and signaling complexes. Activated PI3K promotes PIP2 phosphorylation to produce PIP3, which activates PDK1. AKT is then phosphorylated at Thr308 of PDK1. Ultimately, AKT induces cell proliferation, differentiation, migration, and angiogenesis by mediating multiple signaling pathways such as mTOR, GSK-3β, P53, CREB, P27, and NF-κB.

The PI3K/AKT signaling pathway is involved in regulating cell adhesion, growth, survival, migration, and other cellular events (73). PI3K is an intracellular lipid kinase that affects the expression levels of extracellular protein kinase and EGFR, leading to PIP2 phosphorylation to produce PIP3. PIP3 is an important messenger that recruits AKT, which in turn generates mammalian targets of rapamycin (mTOR) or GSK-3β signaling, resulting in a variety of cellular events (74). The PI3K/AKT/mTOR signaling pathway is one of the most critical abnormal regulatory pathways in CRC, and the activation of this pathway is associated with cell proliferation and transformation, tumorigenesis, progression, angiogenesis, and drug resistance (75, 76) (**Figure 3**).

With the continuous development of high-throughput sequencing technology, a large number of circRNAs with abnormal expression in a variety of tumors have been identified (60, 77, 78). The researchers concluded that the expression level of circ-0008285 in CRC tissues and cells was negatively correlated with tumor size and lymphatic metastasis by combining quantitative reverse transcription-PCR (RT-qPCR) and clinicopathological parameter analysis. Further functional and mechanistic studies confirmed that low expression of circ_0008285 promotes the proliferation and migration of CRC cells *in vitro* by regulating the PI3K/AKT pathway (79). In addition, Jiang et al. found a circRNA that also plays a role in the occurrence and progression of CRC by regulating the PI3K/AKT signaling pathway, named circ-IL4R. However, circIL4R is highly expressed in the serum, tissues, and tumor cell lines of CRC patients, and is positively associated with later clinical stages and poorer prognosis (80).

### CircRNA/VEGF signaling axis in CRC

**Figure 4:**
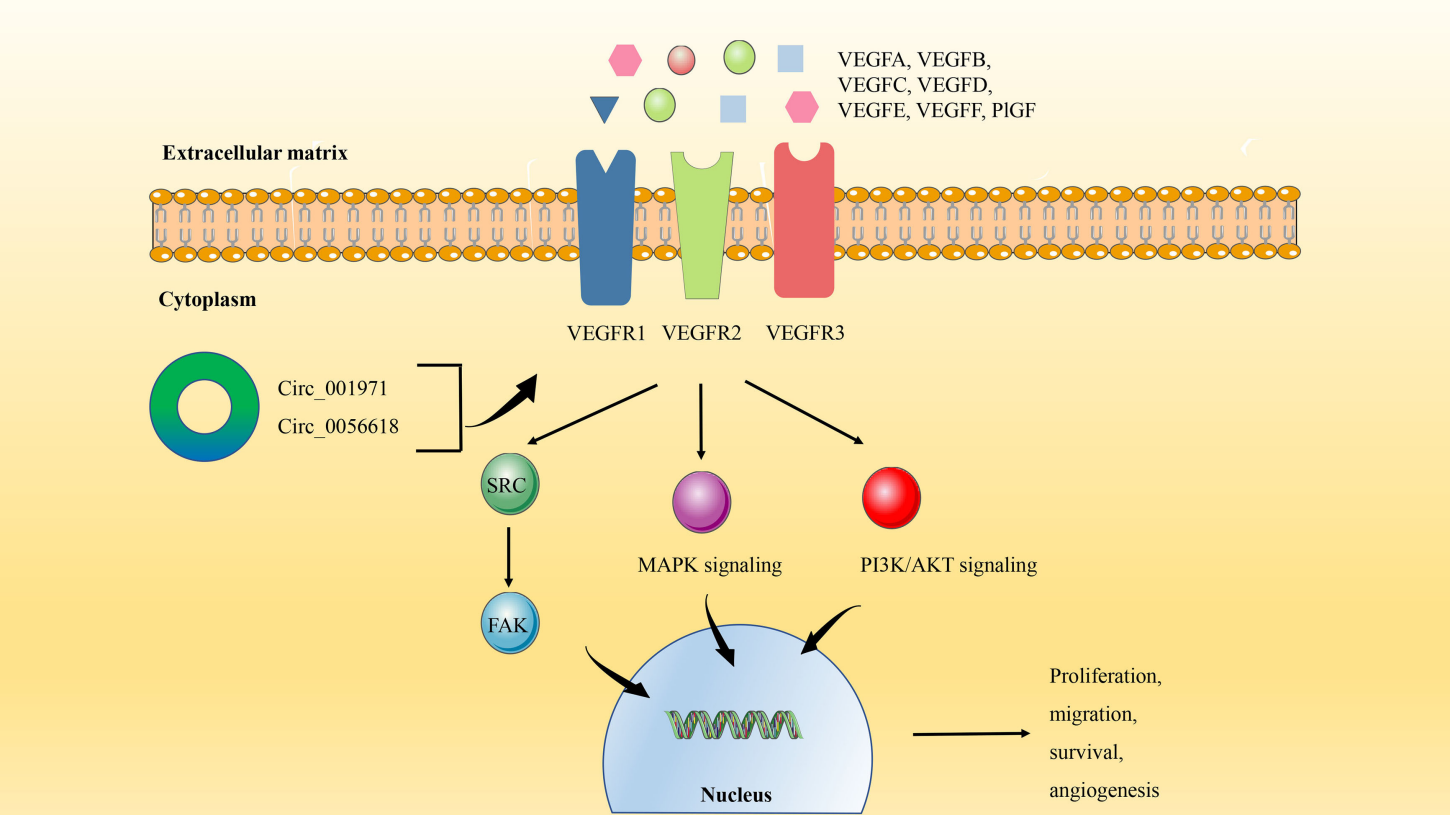
The circRNA/VEGF signaling axis in CRC. VEGF binds to tyrosine kinase cell receptors (VEGFR1-3) to activate VEGF signaling in endothelial cells. The activation of VEGF signaling can not only induce the protein expression of SRC kinase and FAK Focal adhesion kinase, but also trigger PI3K/AKT and MAPK signal transduction. Finally, it regulates the process of angiogenesis, proliferation, migration, and survival.

Generally, the most common distant metastasis site of CRC is the liver. According to statistics, about 25% of CRC patients will have liver metastasis, and the prognosis is poor (81). High expression of the VEGF family is often associated with the aggressiveness and metastasis of CRC (82). VEGF protein family includes VEGFA-F and placental growth factor (PlGF). VEGFs bind to tyrosine kinase cell receptors (VEGFR1-3) to activate VEGF signaling in endothelial cells, affecting cell proliferation, migration, survival, and vascular permeability during angiogenesis (83, 84) (**Figure 4**).

VEGFA is often overexpressed in CRC and is considered to be a key factor in inducing tumor angiogenesis, which plays an important role in tumorigenesis, tumor development, and metastasis (85). Besides the previously enumerated signaling pathways, circRNA can also play a tumorigenic role in CRC through the VEGFA signaling axis. Circ_001971 has been observed to act as a ceRNA to mitigate VEGFA inhibition by miR-29C-3p, thereby enhancing the proliferation, invasion, and angiogenesis of CRC (31). In addition, studies have shown that high expression of circ_0056618 not only produces the same effect on CRC by regulating VEGFA as mentioned above, but also is related to the poor overall survival (OS) of CRC patients (86).

### CircRNA/JAK/STAT signaling axis in CRC

**Figure 5:**
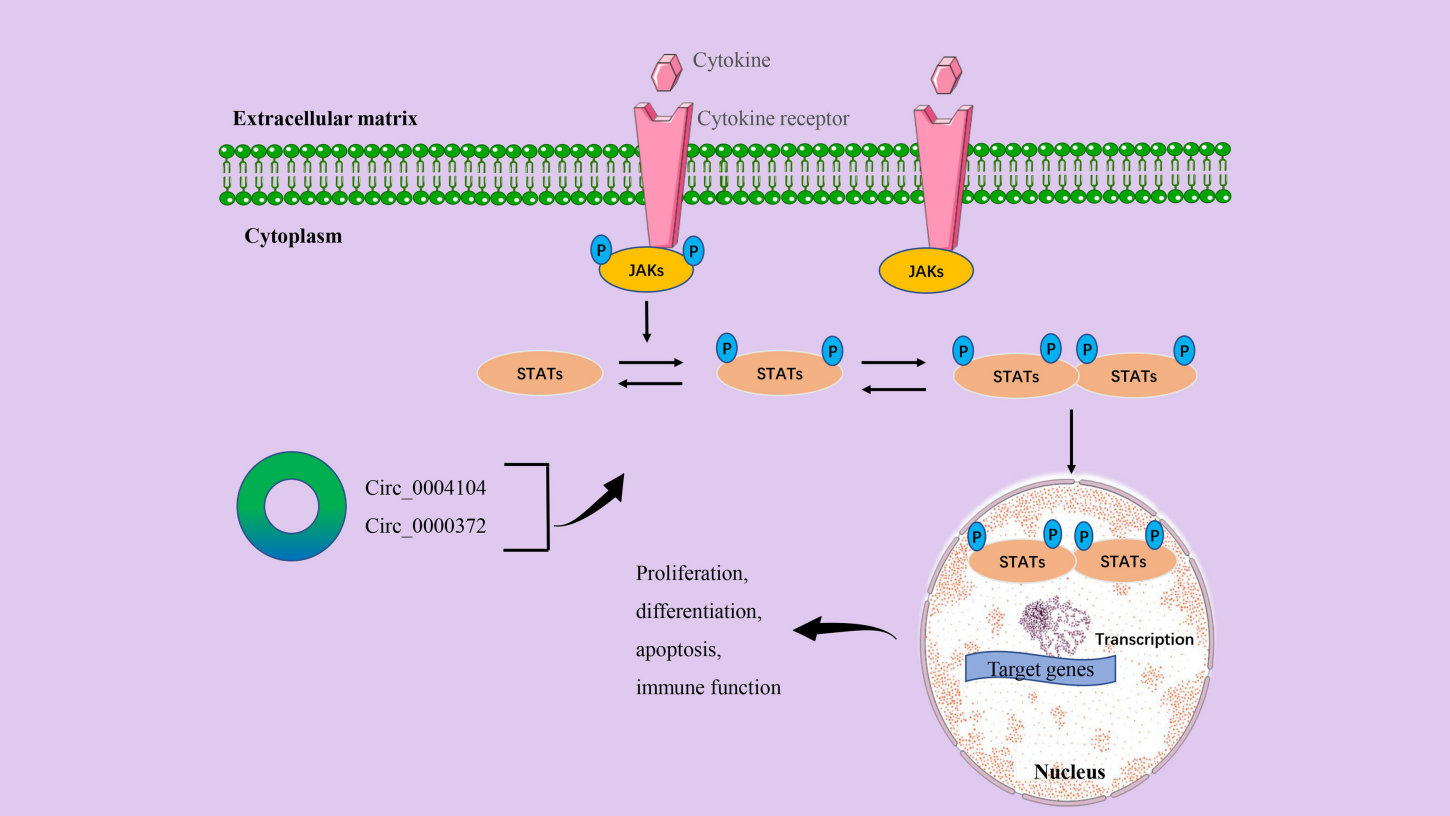
The circRNA/JAK/STAT signaling axis in CRC. The binding of the cytokine to the receptor induces receptor dimers and initiates signal transduction. After JAKs are activated and phosphorylated, STATs proteins are recruited to the phosphorylated tyrosine site. STATs are also then activated and phosphorylated. Normally, STATs reside in the cytoplasm and form phosphorylated dimers when activated by upstream signals. STAT-STAT dimers are transferred from the cytoplasm to the nucleus and regulate the transcription of target genes. Finally, it affects the proliferation, differentiation, invasion, inflammation, and immune function of cancer cells.

The JAK/STAT signaling pathway is a common intracellular signal transduction pathway that is involved in many biological processes such as cell proliferation, differentiation, apoptosis, and immune regulation (87). In CRC, the JAK/STAT signaling axis has been shown to play a key role in tumor cell genesis, progression, invasion, migration, and chemical tolerance (88). The specific molecular mechanisms by which the JAK/STAT signal transduction pathway regulates CRC progression refer to the expression of multiple proto-oncogenes, tumor suppressor genes, cytokines, and their receptors, including Ras, Src, p27^kip1^, p16^ink4a^, interleukin, and EGFR (88, 89). The classic JAK/STAT signaling process is that the connection between the ligand and the receptor activates JAK to form phosphorylation (P)-JAK and forms a docking site for STAT. At this docking site, P-JAK phosphorylates STAT so that it dimers with other members of the STAT family. These dimers will transfer from the cytoplasm to the nucleus and regulate the transcription of target genes (88, 90) (**Figure 5**).

We have listed a variety of circRNAs that play a key role in the occurrence and development of human CRC, but circRNAs alone may not be enough to promote cancer progression. In a study by Wang et al., the initial RNA sequencing found that circ_0004104 expression levels were significantly upregulated in CRC tissues and were closely related to the prognosis of CRC patients. In-depth mechanistic studies have shown that circ_0004104 modulates the JAK2/STAT3 pathway by acting as a ceRNA binding to miR-485-3p and FUS, ultimately promoting cell proliferation and migration (19). Previous studies have demonstrated that the inflammatory cytokine interleukin-6 (IL6) can mediate the activity of the JAK2/STAT3 signaling pathway to participate in the occurrence and development of CRC (91). Recent evidence also shows that down-regulation of circ_0000372 can inhibit the protein expression of the IL6/AK2/STAT3 signaling axis. Further results confirmed that circ_0000372 may regulate IL6 expression and JAK2/STAT3 signaling pathway activity by acting on miR-495 in CRC (28).

### CircRNA/Notch signaling axis in CRC

**Figure 6:**
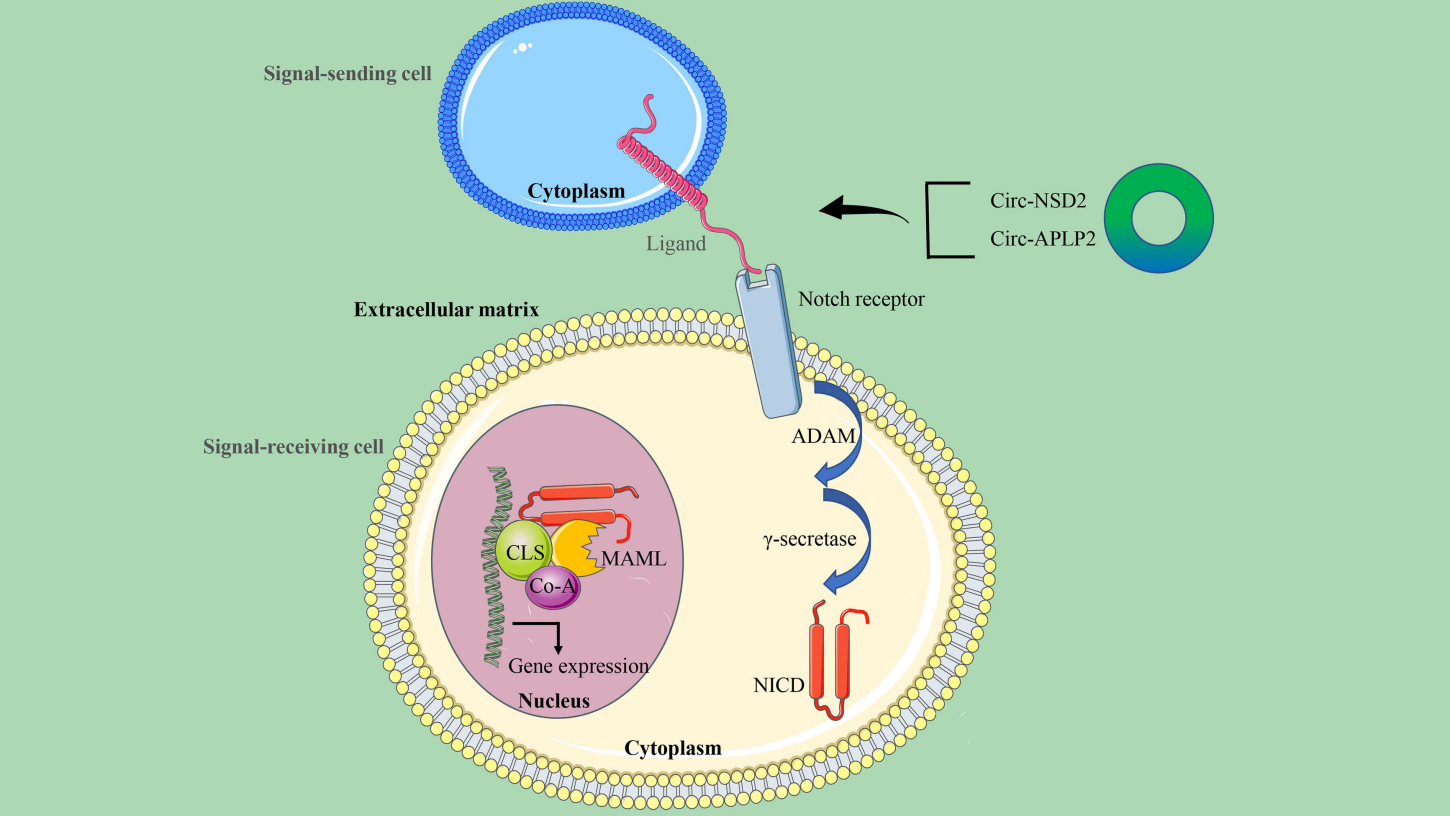
The circRNA/Notch signaling axis in CRC. This signaling is initiated by ligand-receptor binding between the signal-sending cell and the signal-receiving cell. The receptor-ligand interaction triggers a continuous cleavage mediated by ADAM metalloproteinase and γ-secretase, followed by the release of the intracellular domain NICD by Notch. When NICD is transferred to the nucleus, it recruits MAML and Co-A to CSL to initiate the expression of target genes.

There are generally four Notch receptor subtypes (Notch-1, Notch-2, Notch-3, and Notch-4) and five Notch ligands (Dll-1, Dll-3, Dll-4, Jagged-1, and Jagged-2) in humans (92). The Notch signaling pathway, which is involved in the progression of CRC and the self-renewal and homeostasis of normal intestinal epithelium, is activated when the ligand binds to the receptor (93). Abnormal activation of Notch1 has been reported to initiate CRC and enhance its invasiveness (94). The specific mechanism attributed to Notch1 signaling creates a tumor microenvironment (TME) and promotes CRC metastasis through TGF-β-dependent neutrophil recruitment (95) (**Figure 6**).

Tumor metastasis is an important factor affecting the survival and prognosis of patients, and circRNA has been confirmed to be involved in the metastasis of CRC cancer. Using RNA transcriptome sequencing, Chen et al. identified a novel highly expressed circRNA, circ-NSD2, in a mouse model of liver metastasis. A series of functional and mechanistic studies revealed that circ-NSD2 may promote the migration, invasion, and metastasis of CRC cells *in vitro* and *in vivo* by targeting miR-199b-5p mediated JAG1/Notch1 signaling (27). It can also be seen that more and more experimental results reveal that many signaling pathways, including the Notch signaling pathway, are regulated by miRNA. Therefore, based on the results obtained from bioinformatics analysis, miR-101-3p has binding sites on circ-APLP2 and Notch1. Circ-APLP2 has been proven to act as a miR-101-3p sponge to regulate the Notch1 signaling pathway in CRC and activate proliferation and metastasis-related signals (c-Myc, cyclin D1, MMP-2, and MMP-9), thereby promoting the proliferation and liver metastasis of CRC (96).

### CircRNA/Hippo/YAP signaling axis in CRC

**Figure 7:**
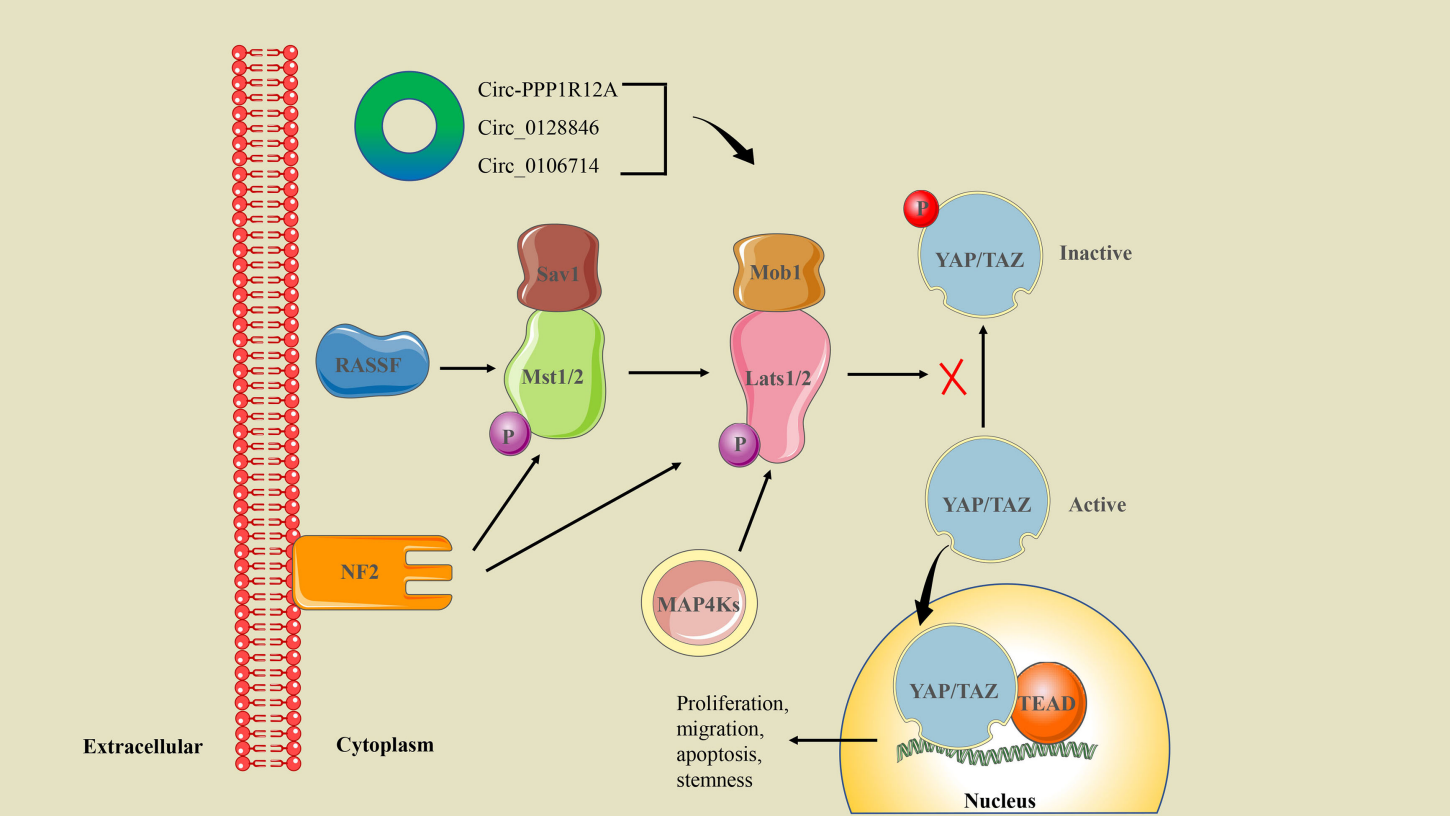
The circRNA/Hippo/YAP signaling axis in CRC. Mst1/2 is activated by upstream NF2 and RASSF family proteins. Phosphorylated Mst1/2 and MAP4Ks transmit multiple signals to activate Lats1/2, which inhibit YAP/TAZ phosphorylation. The activated YAP/TAZ enters the nucleus and binds with the transcription factor TEADs to induce gene expression. Ultimately, it regulates cell proliferation, migration, apoptosis, and the stemness properties of CSCs.

CSCs are the main cause of drug resistance and disease recurrence in CRC treatment. Hippo/YAP is an important signaling pathway involved in the regulation of CSCs, and YAP1 signaling is closely associated with the proliferation and metastasis of CRC cells (58). Hippo pathway core kinases include Mst1/2, Sav1, Lats1/2, and Mob1 (97). When Hippo signaling is activated, the Mst1/2 kinase and Sav1 complex co-phosphorylate and activate Lats1/2 kinase. Subsequently, the downstream transcription coactivators YAP and TAZ are inactivated through cytoplasmic retention and protein degradation, which ultimately regulate the expression of target genes and promote tumor progression (98) (**Figure 7**).

Interestingly, there is growing evidence that circRNA can act as an oncogene or tumor suppressor to regulate the CSC-related Hippo/YAP signaling pathway. Recently, circ-PPP1R12A was screened for elevated expression in colon cancer cytoplasm. Circ-PPP1R12A encodes the conserved 73-aa small peptide PPP1R12A-C (but not circ-PPP1R12A itself), which promotes the proliferation, migration, and metastasis of colon cancer *in vitro* and *in vivo* by activating the Hippo/YAP signaling pathway (51). In addition, other studies have proved that circ_0128846 and circ_0106714 regulate the proliferation and migration of CRC cells through the Hippo/YAP signaling pathway mediated by miR-1184 and miR-942-5p, respectively (29, 46).

### CircRNA/WNT/β−catenin signaling axis in CRC

**Figure 8:**
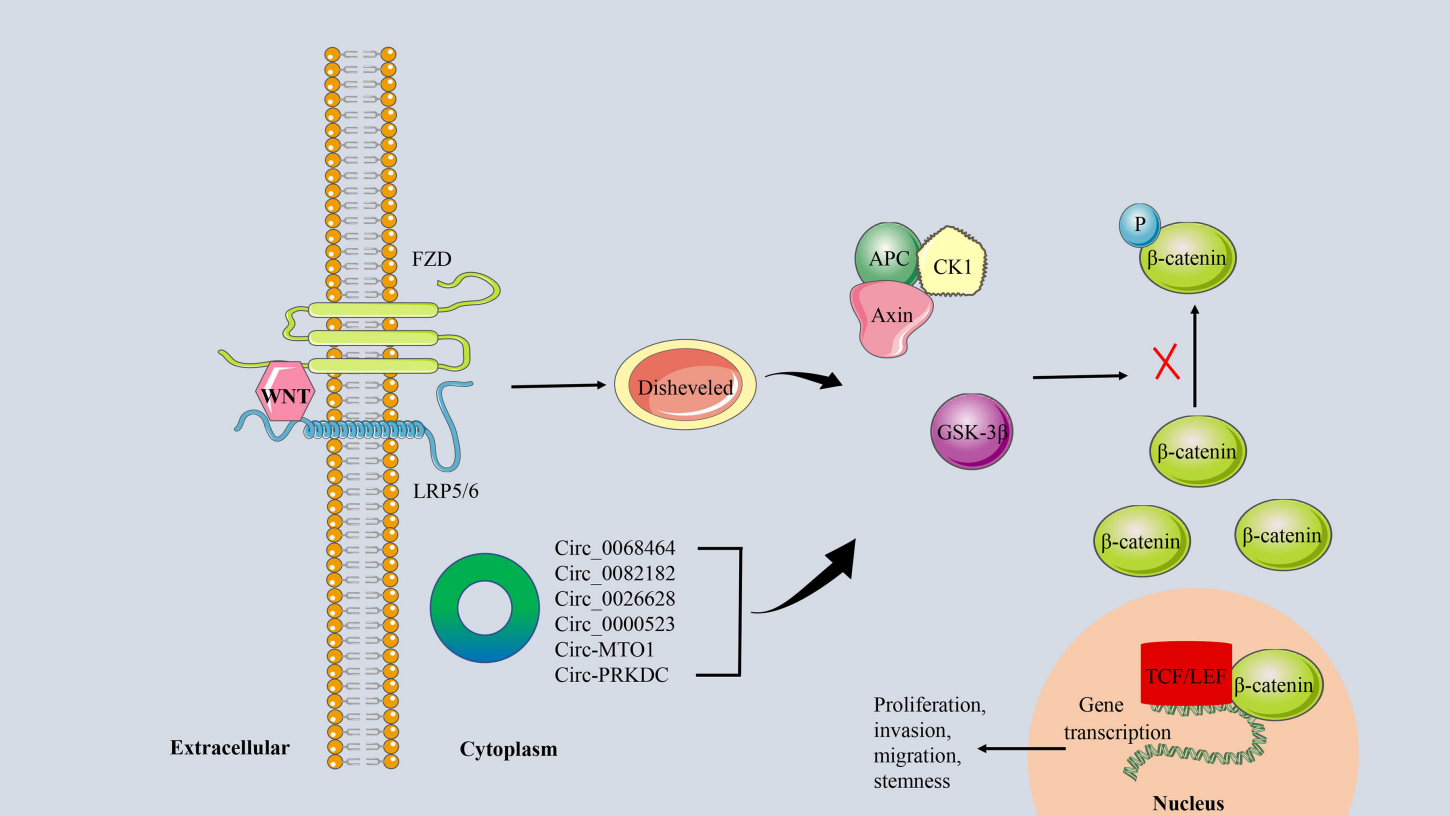
The circRNA/WNT/β−catenin signaling axis in CRC. The linking of WNT protein to the FZD-LRP5/6 receptor complex activates the downstream protein Disheveled. It further promoted the dissociation of GSK-3β from Axin, which inhibited the phosphorylation of β-catenin. Unphosphorylated β-catenin is transferred to the nucleus and binds to the transcription factor TCF/LEF to induce transcription of the target genes.

The WNT/β-catenin signaling pathway is a key regulator of normal intestinal stem cell homeostasis (99). Abnormal activation of this pathway is associated with the invasion, migration, proliferation, and differentiation of CRC cells and is a marker of poor prognosis in CRC patients (100). WNT/β-catenin signaling is initiated by binding the WNT protein to the FZD-LRP5/6 receptor complex. This was followed by activation of Disheveled, which further induced dissociation of GSK-3β from Axin (101). This process prevents the WNT-FZD-Axin-LRP5/6 complex from phosphorylating β-catenin. The accumulation of unphosphorylated β-catenin in the cytoplasm translocates to the nucleus, where it binds to transcription factors such as the TCF/LEF family, resulting in the transcription of target genes that enhance CRC stemness and promote CRC progression (102, 103) (**Figure 8**). Cancer-related deaths in CRC patients are partly due to treatment failure due to chemotherapy resistance. WNT/β-catenin signaling has been shown to mediate chemical resistance to CRC in ncRNA, CSCs, and TME (104).

As a special type of ncRNA, circRNA can also influence tumor progression by regulating the WNT/β-catenin signaling pathway in CRC. For example, circ_0068464 (105), circ_0082182 (106), and circ_0026628 (107), which are highly expressed in CRC, can target corresponding miRNAs to regulate the activity of the WNT/β-catenin pathway, thereby promoting tumor progression. In addition, low expression of circRNA, such as circ_0000523 (108) and circ-MTO1 (109), can also participate in the proliferation and invasion of CRC cells *in vitro* by activating the WNT/β-catenin signaling pathway. 5-FU is a chemotherapeutic drug commonly used in the clinical treatment of CRC, and circRNA has been proven to be a basic regulator of cancer drug resistance (110). Chen et al. found that circ-PRKDC is up-regulated in 5-FU-resistant CRC tissues and cells, and inhibition of circ-PRKDC expression can improve the sensitivity of CRC cells to 5-FU by regulating WNT/β-catenin signaling (111).

## Potential clinical application value of circRNAs signal axis in CRC

### The diagnostic, prognostic, and therapeutic value of circRNAs in CRC

According to statistics, from 2009 to 2015, the 5-year survival rate of CRC in the United States reached 64%, while the survival rate in many Eastern and Southern European countries was less than 50% (112). In particular, the 5-year survival rate for advanced metastatic CRC is only 14% (113). Therefore, it is necessary to find more effective biomarkers for the early diagnosis and treatment of CRC. Studies have shown that the expression of circ_3823 in serum has high sensitivity and specificity for detecting CRC, suggesting that circ_3823 can be used as a potential biomarker for the diagnosis of CRC (114). In addition, the high expression of circ_0004104 in CRC can not only promote cell proliferation and migration but is also closely related to the prognosis of CRC patients, making it a potential therapeutic target for CRC patients (19). Similarly, high levels of circ-MYH9 predict shorter relapse-free survival and OS in CRC patients, so regulation of circ-MYH9 may lead to an effective treatment for CRC (115). Chemotherapy is one of the main treatment methods for CRC, and oxaliplatin is a more commonly used chemotherapy drug. At present, resistance to CRC treatment is still an important issue for controlling the progression of the disease (116). It has been confirmed that glycolysis and resistance of drug-sensitive cells can be enhanced when exosomes present circ_0005963. The results also showed that the expression level of circ_00059633 in serum exosomes was positively related to the chemoresistance of CRC cells to oxaliplatin, and that silencing of the circRNA could reverse the resistance to oxaliplatin (117). More comprehensive clinical application data of circRNAs in CRC is summarized in **Table 1**.

**Table 1:** Potential clinical application value of circRNAs in CRC.

CircRNA	Expression	Effect	Potential application	Reference
Circ_3823	Upregulated	Promote growth, metastasis, and angiogenesis	Diagnostic marker or therapeutic target	(114)
Circ_0004104	Upregulated	Promote proliferation, and migration	Diagnostic and prognostic biomarker, and therapeutic target	(19)
Circ-MYH9	Upregulated	Promote proliferation	Therapeutic target	(115)
Circ_0005963	Upregulated	Promote glycolysis and oxaliplatin resistance	Therapeutic target of drug‐resistant patients	(117)
CircRNA_0001178	Upregulated	–	Diagnosing liver metastases from CRC	(18)
CircRNA_0000826	Upregulated	–	Diagnosing liver metastases from CRC	(18)
Circ-HERC4	Upregulated	Promote proliferation and migration/Induce liver and lung metastasis	Prognostic biomarker and therapeutic target	(20)
Circ-MAPK14	Downregulated	Block progression and metastasis	Therapeutic target	(70)
Circ-ITGA7	Downregulated	Suppress growth and metastasis	Therapeutic target	(71)
CiRS-7	Upregulated	Promote proliferation, migration, and invasion	Prognostic biomarker and therapeutic target	(72)
Circ_0008285	Downregulated	Inhibit proliferation and migration	Therapeutic target	(79)
Circ-IL4R	Upregulated	Promote proliferation, migration, and invasion	Diagnostic and prognostic biomarker, and therapeutic target	(80)
Circ_001971	Upregulated	Promote proliferation, invasion, and angiogenesis	Therapeutic target	(31)
Circ_0056618	Upregulated	Promote proliferation, migration, and angiogenesis	Therapeutic target	(86)
Circ-SPARC	Upregulated	Promote proliferation and migration	Diagnostic and prognostic biomarker, and therapeutic target	(19)
Circ_0000372	Upregulated	Promote proliferation, migration, and invasion	Prognostic biomarker and therapeutic target	(28)
Circ-NSD2	Upregulated	Promote migration and metastasis	Prognostic biomarker and therapeutic target	(27)
Circ-APLP2	Upregulated	Promote proliferation, migration, and invasion/Induce tumor growth and liver metastases	Therapeutic target	(96)
Circ-PPP1R12A	Upregulated	Promote proliferation, migration, and invasion	Therapeutic target	(51)
Circ_0128846	Upregulated	Promote proliferation, migration, invasion, and cell cycle progression/Inhibit apoptosis	Therapeutic target	(29)
Circ_0106714	Downregulated	Inhibit proliferation, migration, and invasion/Promote apoptosis	Prognostic biomarker and therapeutic target	(46)
Circ_0068464	Upregulated	Promote proliferation, migration/Induce tumor growth and lung metastasis	Diagnostic biomarker and therapeutic target	(105)
Circ_0082182	Upregulated	Promote proliferation, cell cycle progression, and metastasis/Inhibit apoptosis.	Diagnostic biomarker and therapeutic target	(106)
Circ_0026628	Upregulated	Promote proliferation, migration, EMT, and stemness	Therapeutic target	(107)
Circ_0000523	Downregulated	Inhibit proliferation/Promote apoptosis	Therapeutic target	(108)
Circ-MTO1	Downregulated	Inhibit proliferation and invasion	Therapeutic target	(109)
Circ-PRKDC	Upregulated	Enhance 5-FU resistance	Therapeutic target of 5-FU-resistant patients	(111)

## Clinical application of circRNAs related signaling pathways in CRC

Early CRC patients are still mainly treated with surgery, and mCRC patients are based on chemotherapy and targeted therapy. Activation of RAS and BRAF mutations in MAPK signaling is one of the most common mutations in human tumors, and the presence of BRAF^V600E^ mutations is considered to be a marker of poor prognosis in mCRC patients (66). Dabrafenib, encorafenib, and vemurafenib (inhibiting BRAF signaling), and trametinib and binimetinib (inhibiting MEK signaling) have previously been developed for the treatment of mCRC by blocking the MAPK pathway (118). However, some mutations such as KRAS and BRAF make mCRC resistant to these therapies (61). Some preclinical data suggest that abnormal activation of EGFR, PI3K signaling, and WNT signaling pathways may be responsible for resistance to BRAF inhibitor monotherapy in patients with BRAF^V600E^ mCRC (35, 119, 120). Therefore, preclinical studies have been conducted, such as BRAF inhibitor and anti-EGFR antibody dual therapy (vemurafenib + panitumumab) (121), BRAF inhibitor and MEK inhibitor dual therapy (dabrafenib + trametinib) (122), and BRAF inhibitor, anti-EGFR antibody with PI3K or MEK inhibitor triple therapy (encorafenib, cetuximab, and alpelisib or binimetinib) (123, 124). Moreover, these combination therapies involving MAPK pathway blocking have achieved certain efficacy in BRAF^V600E^ mCRC patients. However, the three-drug combination therapy brought more adverse reactions to patients, such as hyperglycemia, nausea, diarrhea, and so on (123). In addition, studies have shown that circ_0131663 (70), circ-ITGA7 (71), and CIRS-7 (72) affect the progression and metastasis of CRC by regulating the MAPK signaling pathway. We believe that the targeted therapies have certain efficacy in specific circRNA patients. Further research is expected. The above data is presented in detail in **Table 2**.

**Table 2:** Clinical trials of circRNAs related signaling pathways in CRC.

Drug	Signaling pathway	Therapeutic targets	Phase	Reference/NCT number	Identified circRNAs
Encorafenib	MAPK	BRAF inhibitor	Phase I	(118)	Circ_0131663 (70) Circ-ITGA7 (71) CIRS-7 (72)
Vemurafenib	MAPK	BRAF inhibitor	Phase II	NCT00405587	
Trametinib + panitumumab	MAPK	MEK inhibitor and anti-EGFR antibody	Phase II	NCT02399943	
Dabrafenib + trametinib	MAPK	BRAF inhibitor and MEK inhibitor	Phase I/II	(122)	
Dabrafenib + panitumumab	MAPK	BRAF inhibitor and anti-EGFR antibody	Phase I	NCT01750918	
Vemurafenib + cetuximab/panitumumab	MAPK	BRAF inhibitor and anti-EGFR antibody	Phase I/Phase II	(121)	
Encorafenib + cetuximab	MAPK	BRAF inhibitor and anti-EGFR antibody	Phase III	NCT02928224	
Encorafenib + cetuximab + binimetinib	MAPK	BRAF inhibitor, anti-EGFR antibody with MEK inhibitor	Phase III	NCT02928224	
Dabrafenib + trametinib + panitumumab	MAPK	BRAF inhibitor and MEK inhibitor with anti-EGFR antibody	Phase I	NCT01750918	
PX-866 + cetuximab	PI3K/AKT	PI3K pan-inhibitor and anti-EGFR antibody	Phase II	(125)	Circ_0008285 (79) Circ-IL4R (80)
BKM120 (buparlisib)	PI3K/AKT	PI3K pan-inhibitor	Phase I/Phase II	(126)/NCT01833169	
BKM120 + irinotecan/docetaxel	PI3K/AKT	PI3K pan-inhibitor	Phase I/Phase I	NCT01304602/NCT01540253	
BKM120 + panitumumab/paclitaxel/everolimus	PI3K/AKT	PI3K pan-inhibitor and anti-EGFR antibody/mTOR inhibitor/mTOR inhibitor	Phase I/II/Phase III/Phase I	NCT01591421/NCT04338399/NCT01470209	
GDC-0941	PI3K/AKT	PI3K pan-inhibitor	Phase I	NCT00876109	
GDC-0941 + erlotinib	PI3K/AKT	PI3K pan-inhibitor and anti-EGFR antibody	Phase I	NCT00975182	
MEN1611	PI3K/AKT	PI3K Selective-inhibitor	Phase I/Ib	NCT04495621	
MEN1611 + cetuximab	PI3K/AKT	PI3K Selective-inhibitor and anti-EGFR antibody	Phase I	NCT04495621	
KRX-0401	PI3K/AKT	AKT inhibitor	Phase I	(127)	
MK-2206	PI3K/AKT	AKT inhibitor	Phase II	NCT01802320	
GDC-0068	PI3K/AKT	AKT inhibitor	Phase I	NCT01090960	
PF-05212384	PI3K/AKT	PI3K/mTOR inhibitor	Phase I	(128)	
BEZ235	PI3K/AKT	PI3K/mTOR inhibitor	Phase I	(129)	
GDC-0980	PI3K/AKT	PI3K/mTOR inhibitor	Phase I	NCT00854152	
DS-7423	PI3K/AKT	PI3K/mTOR inhibitor	Phase I	NCT01364844	
PKI-587	PI3K/AKT	PI3K/mTOR inhibitor	Phase I	NCT00940498	
XL-765	PI3K/AKT	PI3K/mTOR inhibitor	Phase I	NCT00485719	
XL-765 + erlotinib	PI3K/AKT	PI3K/mTOR inhibitor and anti-EGFR antibody	Phase I	NCT00777699	
Temsirolimus + irinotecan/cetuximab	PI3K/AKT	mTOR inhibitor/anti-EGFR antibody	Phase II/Phase I	NCT00827684/NCT00593060	
Everolimus	PI3K/AKT	mTOR inhibitor	Phase II	NCT00419159/NCT01387880/NCT00337545	
Everolimus + BEZ235	PI3K/AKT	mTOR inhibitor + PI3K/mTOR inhibitor	Phase I/II	NCT01508104	
AZD2014 + paclitaxel	PI3K/AKT	mTORC1/mTORC2 inhibitor	Phase I	NCT02193633	
Encorafenib + cetuximab + alpelisib	MAPK and PI3K/AKT	BRAF inhibitor, anti-EGFR antibody with PI3K inhibitor	Phase I/IIb	(123)	Circ_0131663 (70) Circ-ITGA7 (71) CIRS-7 (72) Circ_0008285 (79) Circ-IL4R (80)
BKM120 + binimetinib	PI3K/AKT and MAPK	PI3K pan-inhibitor and MEK inhibitor	Phase Ib	NCT01363232	
BYL719 + LGX818 + cetuximab	PI3K/AKT and MAPK	PI3K Selective-inhibitor, BRAF inhibitor with anti-EGFR antibody	Phase I	NCT01719380	
MK-2206 + AZD6244	PI3K/AKT and MAPK	AKT inhibitor and MEK inhibitor	Phase II	NCT01333475	
PF-04691502 + PD-0325901	PI3K/AKT and MAPK	PI3K/mTOR inhibitor and MEK inhibitor	Phase I	(130)	
BEZ235 + binimetinib	PI3K/AKT and MAPK	PI3K/mTOR inhibitor and MEK inhibitor	Phase I	NCT01337765	
XL147 + pimasertib/erlotinib	PI3K/AKT and MAPK	PI3K/mTOR inhibitor and MEK inhibitor/anti-EGFR antibody	Phase I	NCT01357330/NCT00692640	
Bevacizumab	VEGF	anti-VEGFA antibody	FDA approved	(131)	Circ_001971 (31) Circ_0056618 (86)
Regorafenib	VEGF	anti-VEGFR antibody	FDA approved	(131)	
Ramucirumab	VEGF	anti-VEGFR antibody	FDA approved	(131)	
Ziv-aflibercept	VEGF	anti-VEGF antibody	FDA approved	(131)	
Napabucasin	JAK/STAT	STAT3 inhibitor	Phase III	(132)	Circ_0004104 (19) Circ_0000372 (28)
Napabucasin + bevacizumab + FOLFIRI (5‐FU, leucovorin, and irinotecan)	JAK/STAT and VEGF	STAT3 inhibitor and anti-VEGFA antibody	Phase I	NCT02641873	Circ_001971 (31) Circ_0056618 (86) Circ_0004104 (19) Circ_0000372 (28)
Ruxolitinib + regorafenib	JAK/STAT and VEGF	JAK1/2 inhibitor and anti-VEGFR antibody	Phase II	NCT02119676	
OMP-52M51	Notch	anti-Notch1 antibody	Phase I	(133)	Circ-NSD2 (27) Circ-APLP2 (96)
RO4929097	Notch	Gamma secretase inhibitor	Phase I	NCT01116687	
OMP131R10	WNT/β-catenin	Wnt-receptor complex inhibitor	Phase I	NCTO2482441	Circ_0068464 (105) Circ_0082182 (106) Circ_0026628 (107) Circ_0000523 (109) Circ-MTO1 (109) Circ-PRKDC (111)
PRI-724	WNT/β-catenin	Wnt-receptor complex inhibitor	Phase I/II	NCT01764477	
Foxy 5	WNT/β-catenin	Wnt-receptor complex inhibitor	Phase I/	NCTO2655952	
LGK974	WNT/β-catenin	Wnt-receptor complex inhibitor	Phase I/II	NCTO2278133	
ETC-159	WNT/β-catenin	Wnt-receptor complex inhibitor	Phase I	NCTO2521844	

Currently, compounds targeting the PI3K/AKT signaling axis are mainly divided into four types: PI3K inhibitors, AKT inhibitors, mTOR inhibitors, and dual PI3K/mTOR inhibitors. Class Ia PI3K pan-inhibitors PX-866 and BKM120 (buparlisib) have shown good anti-tumor effects in preclinical studies of a variety of tumors (126, 134) and some clinical trials in CRC patients are ongoing. These included PX-866 in combination with cetuximab (NCT01252628), BKM120 in combination with panizumab (NCT01591421), and BKM120 in combination with paclitaxel (NCT04338399). However, the results are not satisfactory. In patients with mCRC, the addition of PX-866 to cetuximab failed to improve OS, progression-free survival (PFS), and objective response rates, but resulted in greater toxicity (125). The BURAN Study (NCT04338399) is scheduled for completion in December 2023. Other PI3K inhibitors are specific subtype inhibitors with strong targeted inhibition and low toxicity properties, such as MEN1611 and BYL719. Phase I trials are being conducted in mCRC patients with BRAF mutations (BYL719 and LGX818 [BRAF inhibitor] with cetuximab) (NCT01719380) and CRC patients with PIK3CA mutations (MEN1611 and cetuximab) (NCT04495621) (135). Some AKT inhibitors, such as KRX-0401, have been proven to be effective in patients with mCRC (127), while others, such as MK-2206, are still in clinical trials (NCT01802320) (NCT01333475). In addition, dual PI3K/mTOR inhibitors also showed preliminary tumor regression ability in CRC patients, among which representative ones were PF-05212384 (128), PF-04691502 (130), and NVP-BEZ235 (129). At present, the most common mTOR inhibitors in clinical trials are temsirolimus and everolimus, but the overall efficacy of mTOR inhibition in clinical application is limited except for certain disease-stabilizing effects in patients with refractory mCRC (76). However, these targeted inhibitors may have a better therapeutic effect in circ_0008285 (79) and circ-IL4R (80) patients because these two circRNAs can promote the proliferation and migration of CRC cells by regulating the PI3K/AKT signaling pathway. These clinical applications are also listed in **Table 2**.

The VEGFA-targeted monoclonal antibody bevacizumab is the first targeted agent to be approved for the treatment of patients with mCRC. After that, three anti-angiogenic drugs, regorafenib, ramucirumab, and ziv-aflibercept, were also approved in the mCRC (131). Antiangiogenic agents do not directly target cancer cells, but rather target the TME like immune checkpoint inhibitors (136). Due to the inevitable problem of drug resistance, it is also a hot topic to explore the combination therapy of antiangiogenic drugs and immune checkpoint inhibitors in addition to developing new antiangiogenic drugs. On the other hand, the combination of targeted regulation of circ_001971 and circ_0056618 may reverse drug resistance. Clinical reports of JAK inhibitors or STAT inhibitors in the treatment of CRC are rare. In a Phase III trial of patients with mCRC, there were no significant differences in OS, PFS, or disease control between the STAT3 inhibitor (napabucasin) group and the placebo group. However, OS was significantly prolonged in py-STAT3-positive patients treated with napabucasin (132). Several natural and small-molecule inhibitors that inhibit Notch signaling have been shown to induce apoptosis in CRC cells *in vitro*, but they lack target specificity and efficacy in clinical evaluation (94). In addition, monoclonal antibodies targeting Notch1, such as OMP-52M51, did not show significant antitumor efficacy in phase I dose-escalation trials (NCT01778439) (133). Similarly, CRC therapies targeting WNT/β-catenin signaling include natural compounds, small molecules, and biological agents. The drugs currently in clinical trials include vitamin D3, curcumin, genistein, resveratrol, LGK974, and ETC-159 (100). Targeting WNT signaling pathways in CRC seems to be a long and difficult process, and no drugs targeting WNT pathways have been approved at present. These targeted therapies may be beneficial for CRC patients in some identified circRNA patients. The clinical data and potentially identified circRNAs are summarized in **Table 2**.

## Conclusions and perspectives

In conclusion, CRC is still a disease with high morbidity and mortality worldwide. Although with the improvement of people’s health awareness and the continuous improvement of diagnosis and treatment technology, some patients can be detected and treated early with a good prognosis. However, the current treatment methods for patients with advanced metastatic CRC are limited and the prognosis is poor. Therefore, human beings have never stopped exploring the mechanism of CRC occurrence and metastasis. Excitingly, a novel ncRNA, circRNAs, has become the focus of CRC research due to its critical regulatory role in cancer-related processes such as tumorigenesis, development, and apoptosis. To improve patients’ OS, many circRNAs are being developed as potential biomarkers for clinical diagnosis and prognosis of CRC, as well as effective therapeutic targets. More and more studies have shown that circRNA usually promotes the proliferation and metastasis of CRC cells by regulating several important signaling pathways, including PI3K/AKT, MAPK, Notch, JAK/STAT, Hippo/YAP, WNT/β-catenin, and VEGF. Currently, there are very few targeted drugs based on these signaling pathways for the clinical treatment of mCRC patients. This may be related to the lack of a deeper and comprehensive understanding of the biological functions and carcinogenic mechanisms of circRNAs and related signaling pathways in CRC. Therefore, based on the comprehensive elaboration and exploration of the molecular mechanism of CRC occurrence and progression in this review, more circRNA signal axes can be developed as effective targets for clinical diagnosis, prognosis, and treatment of CRC in the future to serve patients.

## Author contributions

SW and LC were mainly responsible for literature review and manuscript writing. HW and GL completed the construction pictures and tables. SW designed the ideas of this paper and modified the final manuscript. All authors contributed to the article and approved the submitted version.

## Funding

This work was supported by : Wuxi Traditional Chinese Medicine Hospital Inheritance Studio construction project (2020 No.5).

## Conflict of interest

The authors declare that the research was conducted in the absence of any commercial or financial relationships that could be construed as a potential conflict of interest.

## Publisher’s note

All claims expressed in this article are solely those of the authors and do not necessarily represent those of their affiliated organizations, or those of the publisher, the editors and the reviewers. Any product that may be evaluated in this article, or claim that may be made by its manufacturer, is not guaranteed or endorsed by the publisher.

## Abbreviations

CRC, colorectal cancer; CSCs, cancer stem cells; circRNAs, circular RNAs; CMS, consensus molecular subtypes; ceRNA, competitive endogenous RNA; EGFR, epidermal growth factor receptor; EMT, epithelial-mesenchymal transition; IL6, interleukin-6; JAK, janus kinase; MAPK, mitogen-activated protein kinases; mCRC, metastatic CRC; mTOR, mammalian targets of the rapamycin; miRNA, microRNA; ncRNAs, non-coding RNAs; OS, overall survival; PlGF, placental growth factor; P-JAK, phosphorylation-JAK; PI3K, phosphatidylinositol 3-kinase**;** PFS, progression-free survival; RT-qPCR, quantitative reverse transcription-PCR; STAT, signal transducers and activators of transcription; TGF-β, transforming growth factor-beta; TME, tumor microenvironment; VEGF, vascular endothelial growth factor.
